# Combined Transcriptomic and Metabolomic Analysis of Women with Polycystic Ovary Syndrome

**DOI:** 10.1155/2022/4000424

**Published:** 2022-08-28

**Authors:** Ye Tian-Min, Lin Suxia, Ding Shufang, Cao Dandan, Luo Long-Dan, Yeung William Shu Biu

**Affiliations:** ^1^Center of Reproductive Medicine, The University of Hong Kong-Shenzhen Hospital, Shenzhen, 518000 Guangdong, China; ^2^Shenzhen Key Laboratory of Fertility Regulation, The University of Hong Kong-Shenzhen Hospital, Shenzhen 518000, China

## Abstract

**Background:**

Polycystic ovary syndrome (PCOS) is a complex class of endocrine disorders with insulin resistance, compensatory hyperinsulinemia, and obesity. However, the pathogenesis and therapies of PCOS have not been fully elucidated. Exosomal miRNAs have the potential to serve as biomarkers and therapies for a wide range of medical conditions.

**Method:**

We collected follicular fluid from 5 PCOS patients and 5 healthy people. High-throughput sequencing technology to identify differentially expressed miRNAs and untargeted metabolome identify differential metabolites in follicular fluid exosomal. RT-qPCR and AUC analysis were performed.

**Result:**

miRNA high-throughput sequencing identified 124 differential miRNAs. RT-qPCR analysis confirmed the sequencing results. These differential miRNA target genes are mainly involved in metabolic pathways. Metabolomics studies identified 31 differential metabolites. miRNA and lncRNA coexpression networks in metabolic pathways rigorously screened 28 differentially expressed miRNAs. This network would identify miRNA signatures associated with metabolic processes in PCOS. Meanwhile, the area under curve of receiver operating characteristic revealed that hsa-miR-196a-3p, hsa-miR-143-5p, hsa-miR-106a-3p, hsa-miR-34a-5p, and hsa-miR-20a-5p were potential biomarkers for the diagnosis of PCOS.

**Conclusion:**

Collectively, these results demonstrate the potential pathogenesis of PCOS, and follicular fluid exosomal miRNAs may be efficient targets for the diagnosis and treatment of PCOS in long-term clinical studies.

## 1. Introduction

Polycystic ovary syndrome (PCOS) is a complex class of endocrine disorders with an overall incidence of approximately 5-20% and a prevalence of 5.61% in Chinese women aged 19-45 years [1, 2]. PCOS is often complicated with hyperinsulinemia, dyslipidemia, and obesity, as well as hypertension, heart disease, and endometrial cancer [3]. At present, the pathogenesis of PCOS has not been fully elucidated, and there is a lack of precise treatment. Insulin resistance and compensatory hyperinsulinism lead to metabolic dysfunction, which significantly contributes to the pathogenesis of PCOS [4]. High expression of androgen is also one of the causes of PCOS. The main cause of hyperandrogenesis is an increase in testosterone, androgen, and dehydroepiandrosterone sulfate. Abnormal and immature oocytes exposed to high levels of androgen in the follicular fluid block the development of dominant follicles, stop the growth of follicles, and even block the growth of follicles [5]. Furthermore, the endometrium is not resistant to progesterone due to the stimulation of estrogen levels, which increases the risk of endometrial cancer [6]. Increasing evidence suggests that PCOS may be a complex polygenic disorder with a strong epigenetic influence. Eleven susceptibility loci were found in Chinese women with polycystic ovary syndrome. Some of these genes, such as INSR, FSHR, and c9orf3, have been identified [7, 8]. Mendelian random analysis showed that single-nucleotide polymorphisms associated with the risk of polycystic ovary syndrome had a causal relationship with higher body mass index (BMI), insulin resistance, and lower levels of sex hormone-binding globulin (SHBG) in patients with polycystic ovary syndrome. Other previously reported gene mutations, namely, in YAP1, THADA, and FSHB, have also been observed to have genome-wide significance [9]. It is worth noting that, to date, the heritability of PCOS may not exceed 10% [10]. Therefore, it is urgent to explore the characteristics and possible pathogenesis of PCOS from different aspects.

Exosomes are key mediators in different physiological and pathological processes and have played increasingly important roles [11]. S100 calcium-binding protein A9 (s100-a9) is enriched in PCOS follicular fluid, and it can significantly enhance inflammation and destroy steroid production by activating the nuclear factor-*κ*b (NF-*κ*b) signaling pathway [12]. The expression of DENND1A variant 2 mRNA was significantly increased in urine exosomes from women with PCOS compared with normal cycling women [13]. Exosomal miR-323-3p from adipose mesenchymal stem cells promoted proliferation and inhibited the apoptosis of cumulus cells in a letrozole-induced PCOS mouse model [14]. During the cellular inflammatory response, the composition of exosomal miRNAs is different from that of normal exosomes [15]. It has been reported that the differential expression of plasma exosomal miRNAs may be related to the occurrence of PCOS and help to differentiate PCOS patients from controls. These results may contribute to the understanding of epigenetic modifications in PCOS pathophysiology [16]. However, there are few studies on miRNAs in PCOS follicular fluid exosomes. The main purpose of this study was to explore the expression profile of miRNAs in PCOS follicular fluid exosomes and to analyze their potentially important role in the development of PCOS.

## 2. Materials and Methods

### 2.1. Sample Collection

This study was approved by the Medical Research Ethics Committee of Shenzhen Hospital, the University of Hong Kong (Ethics Committee of Shenzhen Hospital, HKU) (NO.2018-190). All ethics procedures conformed with the principles of the 1964 Declaration of Helsinki and its latest 2008 amendments. The research was conducted with the informed consent of each participant, and they all signed informed consent forms. Both the PCOS patients and non-PCOS patients consented for sample collection and molecular testing were approved by the University of Hong Kong-Shenzhen Hospital Research Ethics Committee. All investigations were conducted in accordance with the Helsinki Declaration.

### 2.2. Exosomes Isolation and Characterization

Exosomes were isolated from 1-mL follicular fluid using System Bioscience (SBI) ExoQuick™ Exosome Precipitation Kit, according to the supplier's protocols. Transmission electron microscopy was used to detect exosomes size and characterization. In short, a copper mesh was placed on a clean wax plate, and 100 *μ*L of the exosome suspension was added. After 4 minutes, the copper mesh was removed and placed in 2% phosphotungstic acid for 5 min. The mesh was laid on the filter paper to dry, and TEM was used to observe the morphological features of the exosomes [17]. The exosome pellet was dissolved in the protein lysis buffer, and the protein concentration was determined using a Bradford protein assay kit (Bio-Rad Laboratories, Hercules, CA). Western blotting was used to check the marker of exosome via HSP70 and TSG101 primary antibodies (Abcam, Cambridge, UK) (antibody information is shown in Supplementary Table 1).

### 2.3. RNA Isolation from Exosomes

Total RNA was isolated from 200 *μ*L of exosomes suspension using TRIzol reagents (Invitrogen, USA). RNA concentration was measured by Nanodrop 2000 spectrophotometer (Thermo Scientific) and stored at −80°C. All solutions were prepared in RNase-free water, and all methods were carried out in RNase-free conditions.

### 2.4. miRNA Sequencing and Bioinformatics Analysis

miRNA sequencing libraries were constructed by TruSeq Small RNA Library Prep Kit (Illumina) following the manufacturer's instructions. Sequencing libraries were sequenced with a NextSeq apparatus to generate ~16 million single-end 75 bp reads per sample. Afterwards, sequencing reads were obtained the final counts of miRNAs present in each sample. Briefly, adapter sequences were removed from sequencing reads, and the remaining sequences were compared against the human mature miRNA from miRbase (release 22.1) (https://www.mirbase.org/) using FANSe3 for miRNA identification, annotation, and quantification. Differential miRNA expression analysis (*P* < 0.05, log2|*FC*| > 1) between groups of interest was carried out with the R package EdgeR. To predict the genes targeted by differential miRNAs, miRTarBase (https://mirtarbase.cuhk.edu.cn/~miRTarBase/miRTarBase_2022/php/index.php) was used to identify miRNA-binding sites. In addition, Kyoto Encyclopedia of Genes and Genomes (KEGG) pathway and gene ontology (GO) and pathway analyses were performed to identify miRNA-related genes, pathways, and GO terms based on sequencing data sets. Cytoscape (http://www.cytoscape.org/) was used to draw a miRNA-lncRNA network, and the data output was received in Excel spreadsheets.

### 2.5. RT-qPCR Analysis

Total miRNAs from the follicular fluid exosomes were extracted using the TRIpure total RNA extraction reagent method (ELK Biotechnology, China). Real-time PCR was performed for validation using Mir-X miRNA qRT-PCR TB Green® Kit (Takara, Kyoto, Japan). In a simple, single-tube reaction, RNA molecules are polyadenylated and reverse transcribed using poly(A) polymerase. The relative microRNA levels were normalized to U6 expression for each sample. The miRNA primers used in the study are presented in Table 1. The reactions were performed with a Step One Plus Real-Time PCR System (Applied Biosystems) and Step One software v2.1. The PCR reaction included a fast start step of 10 min at 95°C followed by 45 cycles of amplification where each cycle consisted of denaturation at 95°C for 10 s, 58°C for 30 s, and 72°C for 30 s. Analyses of gene expression were performed by the he delta-delta Ct method. Each experiment was repeated three times.

### 2.6. Metabolite Extraction

After the exosome samples were slowly thawed at 4°C, an appropriate amount of the sample was added to precooled methanol/acetonitrile/water solution (2 : 2 : 1, v/v), mixed by vortex, sonicated at low temperature for 30 min, and left at -20°C for 10 minutes. Min, centrifuge at 14,000*g* for 20 min at 4°C, take the supernatant and dry it in vacuo, add 100 *μ*L of acetonitrile aqueous solution (acetonitrile: water =1 : 1, v/v) to reconstitute, vortex, and centrifuge at 14,000*g* for 15 min at 4°C, take the supernatant for injection analysis.

### 2.7. Nontargeted Metabolomics Analysis

The metabolomic profiling was carried out on a UHPLC-Q-TOF MS system (Agilent, CA, USA) with a HILIC column (Agilent, CA, USA). The column conditions were set to include a column temperature of 25°C, a flow rate of 0.5 mL/min, and an injection volume of 2 *μ*L. The samples were separated by an Agilent 1290 Infinity LC ultrahigh performance liquid chromatography system (UHPLC) and analyzed by a Triple TOF 6600 mass spectrometer (AB SCIEX) using electrospray ionization (ESI) positive and negative ion modes, respectively. The raw data were converted into. mzXML format by ProteoWizard and then XCMS software was used for peak alignment, retention time correction, and peak area extraction. The data extracted by XCMS were firstly subjected to metabolite structure identification, data preprocessing, and then to experimental data quality evaluation, and finally to data analysis. Data analysis includes univariate statistical analysis, multidimensional statistical analysis, differential metabolite screening, differential metabolite correlation analysis, and KEGG pathway analysis.

### 2.8. Statistical Analysis

Data are shown as the means ± standard deviations. The statistical significance of the results from three independent assays was evaluated by Student's *t*-test. *P* < 0.05 was considered to indicate statistically significant differences.

## 3. Result

### 3.1. Comparison of Clinical Information between the PCOS Group and the Control Group

To ensure the reliability of the results of this study, the selected samples of this study were strictly screened according to the Rotterdam criteria (2003). There were two groups (PCOS patients =15, control patients =15) of patients, and their follicular fluid exosome samples were analyzed. All enrolled participants were diagnosed with primary infertility and received the same ovulation induction treatment program; they were between 26 and 36 years of age with a duration of infertility between 1 and 5 years. The results showed that there was no significant difference between the PCOS patients and the non-PCOS patients in age, infertility, body mass index (BMI), and fasting blood glucose (FBG) levels. In addition, the number of follicles and anti-Mullerian hormone (AMH) levels were clearly upregulated in the PCOS patients, and there were patients with an LH/FSH>1 in the PCOS group. The general clinical data of the two groups of patients are shown in Table 2.

### 3.2. Identification/Purification of Exosomes Extracted from Follicular Fluid

**Figure 1:**
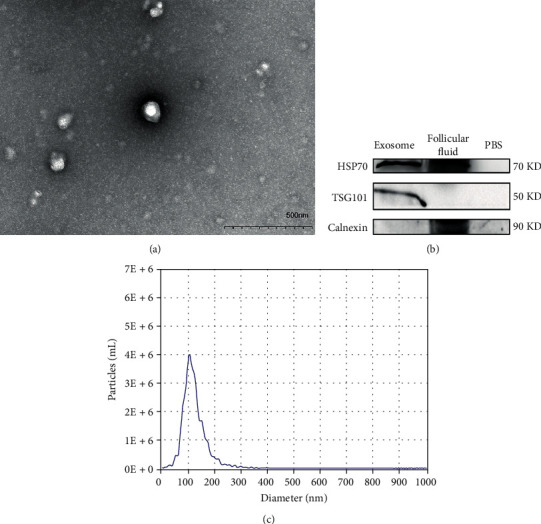
Characterization of exosomes in the follicular fluid exosomes. (a) Isolated exosomes micrograph of TEM. (b) Exosome protein markers validation by western blotting. (c) Nanoparticle tracking analysis the diameter of exosomes.

Exosomes were isolated from follicular fluid. Transmission electron microscopy (TEM) was used to detect exosomes approximately 50–200 nm in diameter from all samples (Figure 1(a)). Western blot analysis was performed and revealed that two commonly used exosomal protein markers, namely, CD9 and TSG101, were highly enriched in the isolated exosomes relative to PBS (Figure 1(b)). Nanoparticle tracking analysis showed the diameter of exosomes (Figure 1(c)). The results showed that exosomes from all follicular fluid samples were successfully purified.

### 3.3. Differential Expression of miRNA Profiles in Follicular Fluid Exosomes

**Figure 2:**
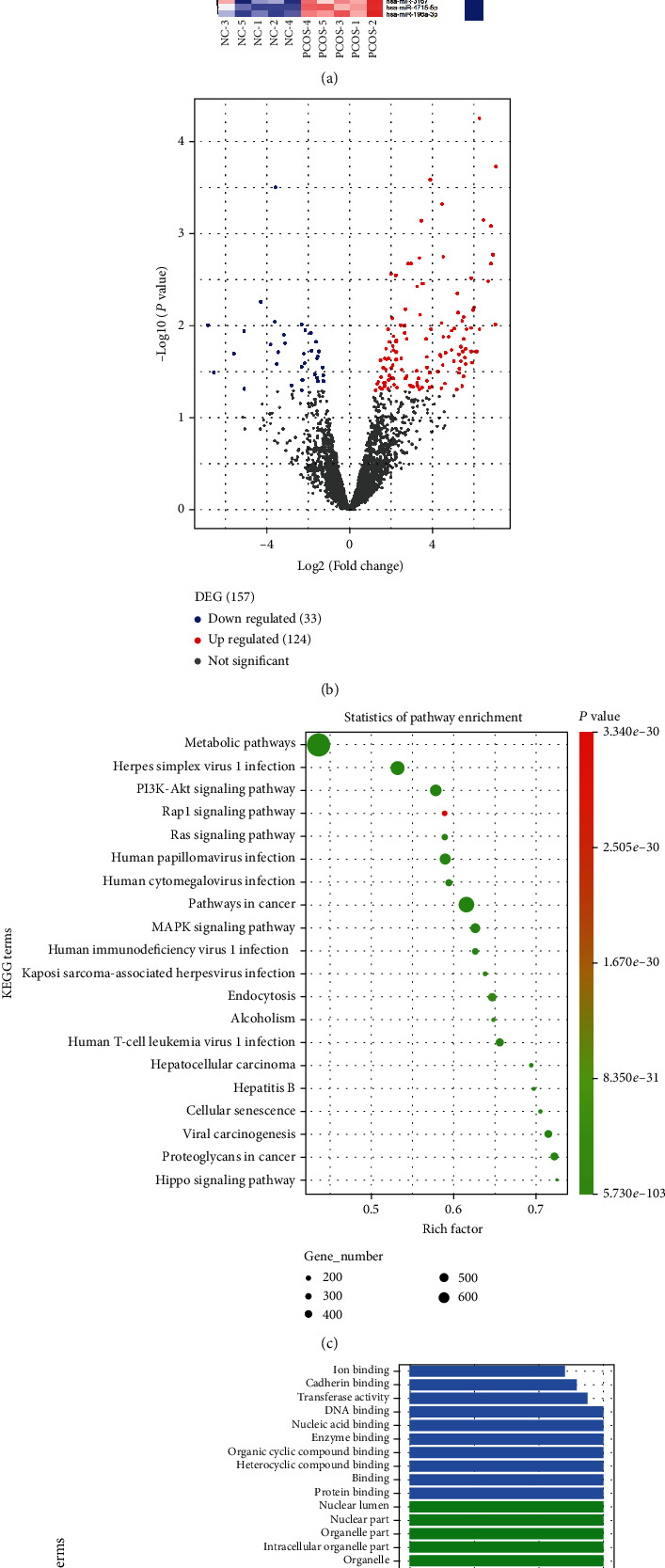
Enrichment analysis of the significantly diff-expressed miRNAs target genes. (a) Heat map of volcano plot of diff-expressed miRNAs between PCOS and NC (non-PCOS). (b) Volcano plot of diff-expressed miRNAs between PCOS and NC (non-PCOS). (c) KEGG pathway enrichment analysis of the significantly diff-expressed miRNAs target genes. (d) GO analysis of the significantly upregulated miRNAs target genes. (e) GO analysis of the significantly downregulated miRNAs target genes.

**Figure 3:**
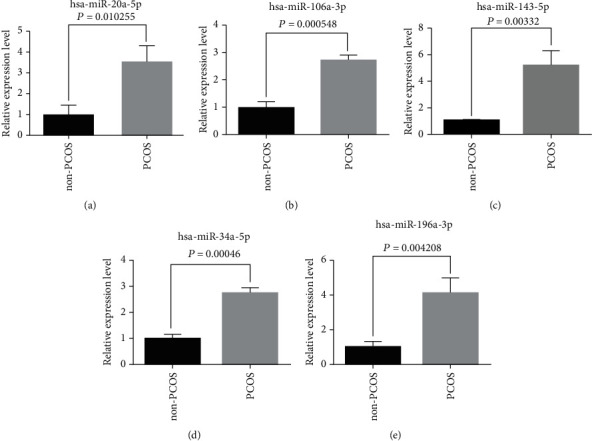
Validation the miRNA profiling by RT-qPCR. means ± standard deviations.

In total, 2,457 miRNAs were identified in follicular fluid exosomes from both PCOS patients and controls in this study. Among them, 157 mature miRNAs in follicular fluid exosomes were significantly differentially expressed in the PCOS and control groups (*P* < 0.05, log2|*FC*| > 1). The number of significantly upregulated miRNAs was 124, and the number of downregulated miRNAs was 33, as indicated by a volcano plot and a heat map (Figures 2(a) and 2(b)). KEGG pathway and GO analyses were performed to investigate the functions of 157 differentially expressed miRNA target genes. Furthermore, KEGG pathway enrichment revealed that the target genes were mainly involved in metabolic pathways (Figure 2(c)). GO enrichment analyses were also carried out to gain insight into the biological characteristics of the miRNAs. Metabolic processes were very prominent in both the significantly upregulated and downregulated miRNA target genes, including nucleic acid metabolic process, cellular macromolecule metabolic process, and heterocycle metabolic process (Figures 2(d) and 2(e)). These results suggest that metabolic pathways possibly have great significance in the pathogenesis of PCOS. To further validate the miRNA profiling results, five miRNAs, hsa-miR-196a-3p, hsa-miR-143-5p, hsa-miR-106a-3p, hsa-miR-34a-5p, and hsa-miR-20a-5p were randomly screened by RT-qPCR from PCOS patients and non-PCOS patients. According to the RT-qPCR results, the expression of the miRNAs determined by RT-qPCR was consistent with those obtained from RNA sequencing in follicular fluid exosome samples between PCOS and non-PCOS (Figure 3).

### 3.4. Construction of the miRNA–lncRNA Coexpression Network

**Figure 4:**
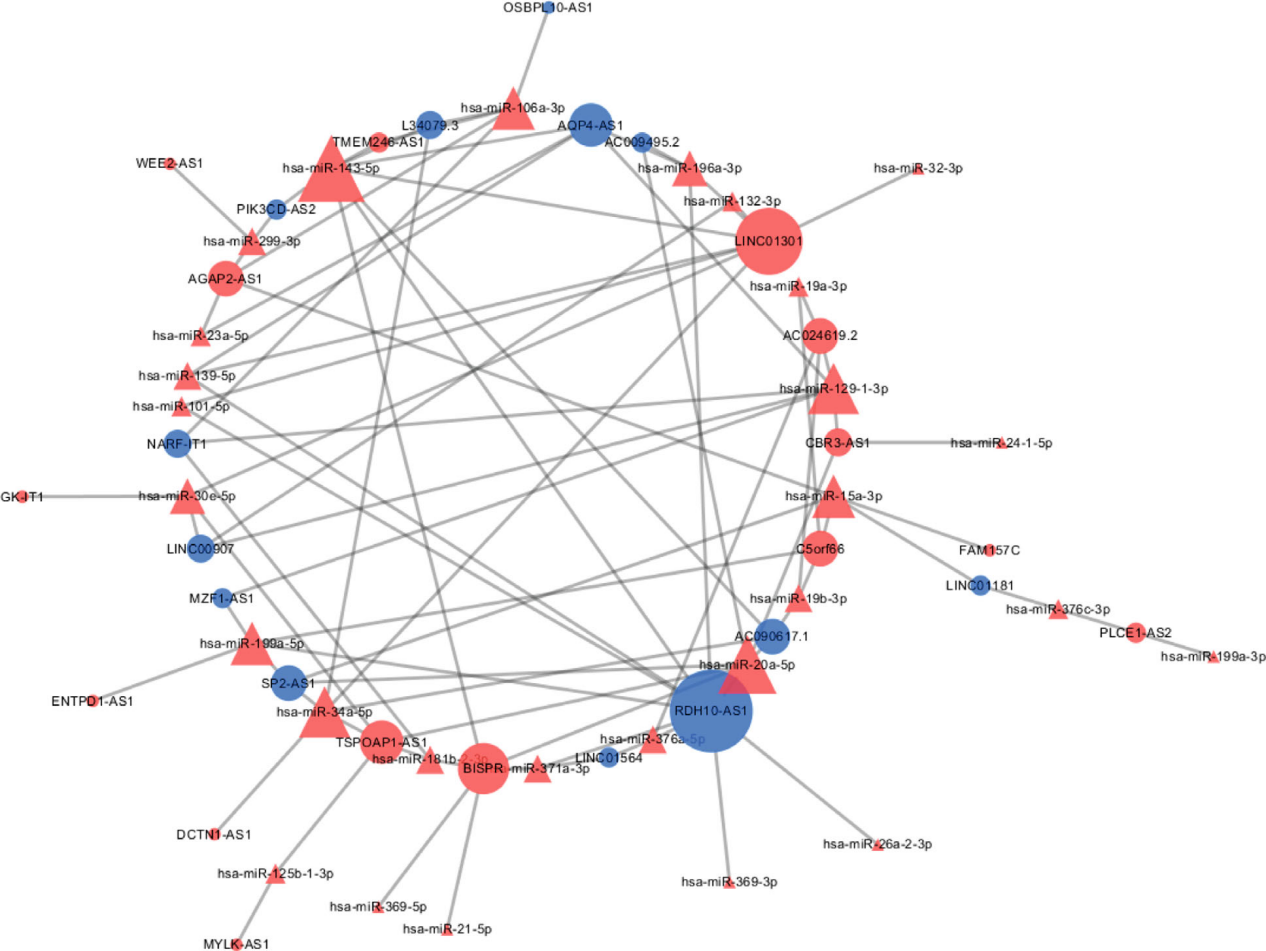
The miRNA–lncRNA coexpression network in metabolic pathways (hsa01100) in follicular fluid exosomes.

**Figure 5:**
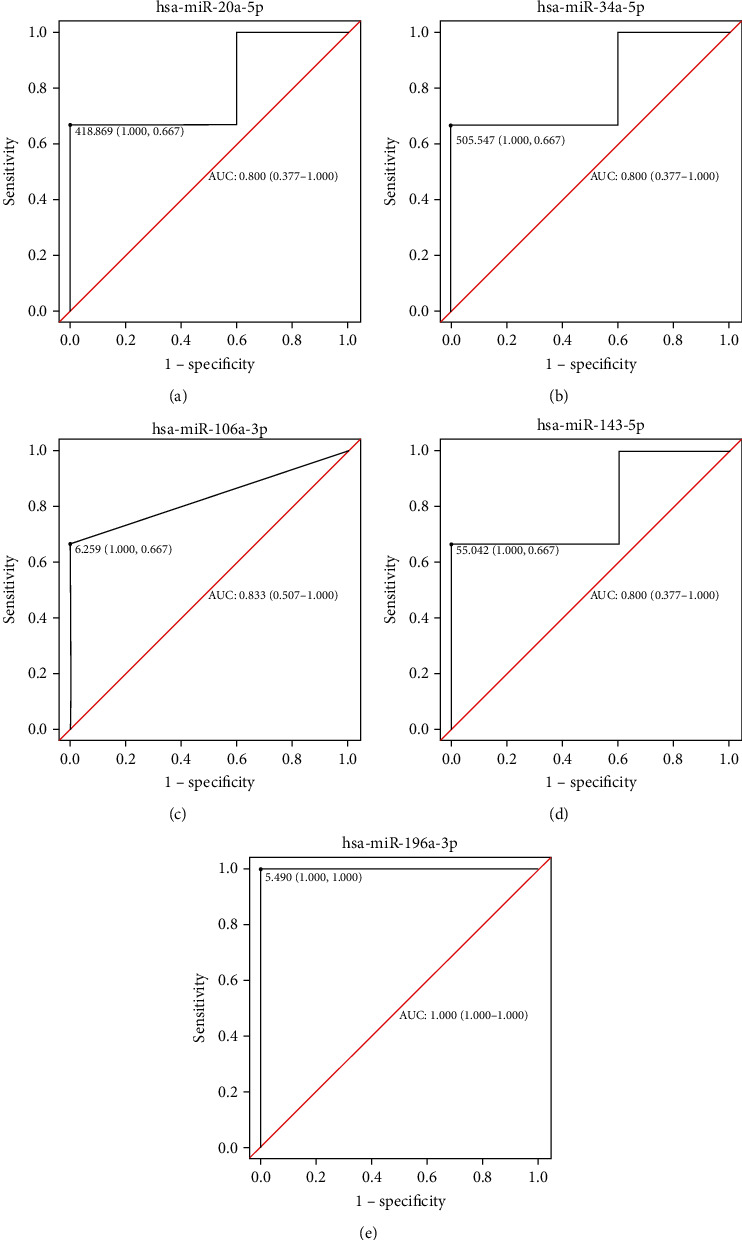
The analysis of the area under curve of receiver operating characteristic of miRNAs.

To further explore the epigenetic regulation of miRNAs, intersection analysis between miRNAs and lncRNAs was performed in follicular fluid exosomes from PCOS and non-PCOS patients. The differentially expressed lncRNAs in this study were strictly screened according to the previous research results of Wang et al. [18]. The genes interacted with lncRNA were mainly enriched in metabolic pathways, which was consistent with our miRNA results. Therefore, a miRNA and lncRNA coexpression network in metabolic pathways (hsa01100) strictly screened 28 differentially expressed miRNAs (Table 3). The results showed that the upregulated miRNAs, such as miR-369-3p, miR-139-5p, miR-371a-3p, miR-143-5p, miR-199a-5p, miR-196a-3p, and miR-26a-2-3p, reduced the expression of RDH10-AS1. In addition, NARF-IT1, AC090617.1, MZF1-AS1, AC009495.2, LINC01564, AQP4-AS1, L34079.3, OSBPL10-AS1, PIK3CD-AS2, LINC01181, LINC00907, and SP2-AS1 were regulated by the differentially expressed miRNAs (Figure 4). Subsequently, to further investigate the specificity and sensitivity of miRNAs involved in the metabolic pathways, the area under curve of receiver operating characteristic were performed to analyze the prognosis of PCOS (Figure 5). The findings suggest that these miRNAs may play a role in the diagnosis and pathogenesis of PCOS.

### 3.5. Differential Metabolites in Follicular Fluid Exosomes between the PCOS Group and the Control Group

**Figure 6:**
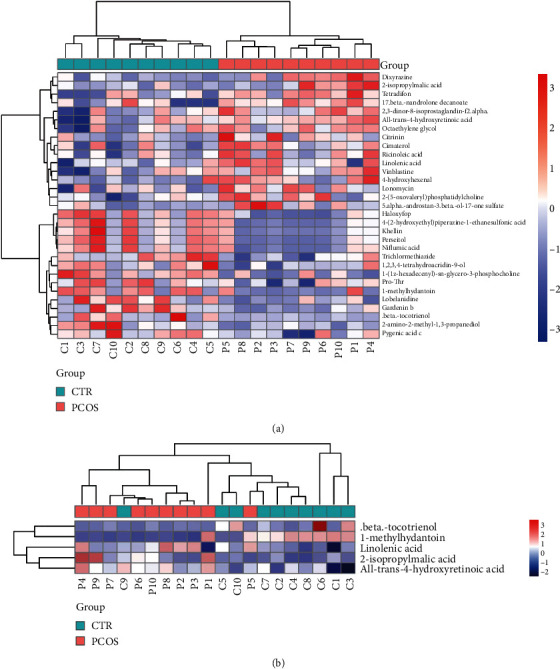
Differential metabolites between PCOS and NC (non-PCOS). (a) Significantly differential metabolite hierarchical clustering heat map. (b) KEGG pathway differential metabolite clustering heat map.

In total, 545 metabolic features were detected with untargeted metabolomics in follicular fluid exosomes from both PCOS patients and controls in this study. OPLS DA VIP>1 and *P* value <0.05 were considered to be significantly different metabolites, and a total of 31 differential metabolites (16 upregulated and 15 downregulated) were identified. Cluster analysis was performed on the screened significantly different metabolites, and the results are shown in Figure 6(a). Metabolites clustered in the same cluster have similar expression patterns and may have similar functions or participate in the same metabolic process or cellular pathway. KEGG pathway was performed to investigate the functions of 31 differentially expressed metabolites. The differential metabolites in the KEGG metabolic pathway with a differential metabolite number greater than 5 are .beta.-tocotrienol, 1-methylhydantoin, 2-isopropylmalic acid, all-trans-4-hydroxyretinoic acid, and linolenic acid (Figure 6(b)).

## 4. Discussion

To date, a variety of factors have been reported to be involved in the pathogenesis and clinical phenotype of PCOS, such as excessive androgen synthesis, follicular atresia, and insulin resistance [19]. However, the causes of PCOS are still unclear. Human follicular fluid contains hormones, growth factors, cytokines, vitamins, and cell metabolites. Some proteins in the follicular fluid are closely involved in glucose metabolism, lipoprotein metabolism, cell proliferation, insulin resistance, and other processes in PCOS patients [20]. Studies suggest that the information carried in follicular fluid is an important entry point for the study of PCOS. A large amount of evidence has shown that the release of membrane-sealed ventricular structures, such as exosomes and extracellular vesicles (EVSs), is an effective mechanism of intercellular communication under normal physiological and pathological conditions. Exosomes and extracellular vesicles in follicular fluid are considered carriers of information. These exosomes may also be involved in the progression of polycystic ovary syndrome and other diseases [21]. Therefore, this study explored the molecular characteristics of exosomal miRNAs in PCOS follicular fluid and elucidated the potential role of these miRNAs by using bioinformatics tools.

PCOS is a multifactorial disease caused by endocrine and metabolic dysfunction, and in recent years, the pathogenesis of polycystic ovary syndrome considered to be related to epigenetics. In cells, miRNAs posttranscriptionally impair mRNA translation and, consequently, a dysregulated miRNA expression profile may affect various cellular processes and pathways, in keeping with the complexity of PCOS [22]. In recent years, more and more evidence suggests that abnormal expression of miRNAs is detected in granulosa cells, theca cells, adipose tissue, follicular fluid, serum, and peripheral blood leukocytes of women with PCOS [23]. Aberrant miRNA expression might be involved in the underlying pathophysiology of PCOS including cell proliferation, apoptosis, steroidogenesis, folliculogenesis, glucose metabolism, and insulin sensitively [24]. In this study, we identified differentially expressed miRNAs in follicular fluid exosomes from PCOS patients and controls. The results from the previous study in plasma were consistent with the results from this study that demonstrated that the expression of exosomal miR-106a-3p in PCOS follicular fluid was downregulated [16]. Previous research has shown that hsa-miR-34a-5p may participate in follicular development and oocyte maturation in PCOS [25, 26]. Elevated serum-free testosterone levels in PCOS patients were significantly associated with miR-20a-5p, and circulating miRNAs may be useful as diagnostic biomarkers in PCOS women with metabolic syndrome [22]. KEGG pathways and GO enrichment analyses revealed that the miRNA target genes were mainly involved in the MAPK signaling pathway and metabolic process. These biological functions have also been found to be associated with the activation of follicular development using lncRNA and mRNA profiles of follicular fluid from mature and immature ovarian follicles of PCOS patients [27]. Interestingly, the expression of exosomal miR-19 and miR-199 was increased in follicular fluid samples from patients with PCOS in this study. In addition, there is strong evidence that the activity and mRNA expression level of CYP19A1 and both miR-19 and miR-199 target genes were decreased in patients with PCOS, and this was associated with decreased follicle size [28, 29]. The differences in expression of miR-196a-3p, miR-143-5p, miR-106a-3p, miR-34a-5p, and miR-20a-5p were consistent with the sequencing results validated by qPCR, and their areas under the curve were all greater than 0.8. These findings suggest that miR-196a-3p, miR-143-5p, miR-106a-3p, miR-34a-5p, and miR-20a-5p may confer a risk of PCOS and may contribute to the diagnosis of PCOS patients and prognosis.

LncRNAs are a class of transcripts (>200 nucleotides) lacking protein-coding capacity, and they function as competitive endogenous RNAs (ceRNAs) and are significantly correlated with some clinical phenotypes in PCOS [30]. Previous studies have found that the expression levels of RDH10-AS1, NARF-IT1, AC090617.1, MZF1-AS1, AC009495.2, LINC01564, AQP4-AS1, L34079.3, OSBPL10-AS1, PIK3CD-AS2, LINC01181, and LINC00907 were reduced in PCOS [18]. MZF1-AS1 has been reported to inhibit proline synthesis and neuroblastoma progression [31]. AC009495.2 was associated with acute myeloid leukemia, and it could differentiate between acute myeloid leukemia types and change the behavior of acute myeloid leukemia cells [32]. Energy stress-induced LINC01564 activated the serine synthesis pathway and facilitated hepatocellular carcinogenesis [33]. AQP4-AS1 plays a potential role in breast cancer [34]. The lncRNA PIK3CD-AS2 promoted lung adenocarcinoma progression via YBX1-mediated suppression of the p53 pathway [35]. In this study, these lncRNAs and differentially expressed miRNAs were used to construct a metabolic pathway-associated lncRNA-miRNA network, which indicated the key mechanisms of PCOS.

PCOS is a metabolic-related disease. In this study, we identified 31 differentially expressed metabolites, among them .beta.-tocotrienol, 1-methylhydantoin, 2-isopropylmalic acid, all-trans-4-hydroxyretinoic acid, and linolenic acid were differential metabolites in the KEGG metabolic pathway with a differential metabolite number greater than 5. Prabhu et al. indicated that gamma-linolenic acid can ameliorate the inflammatory response in DHEA-induced polycystic ovary syndrome via PPAR-*γ* signaling [36]. These five metabolites may be involved in the pathogenesis of PCOS and may be used as a metabolic marker for PCOS. The results of miRNA sequencing revealed that differential miRNAs mainly regulate metabolic pathways. We speculate that miR-196a-3p, miR-143-5p, miR-106a-3p, miR-34a-5p, and miR-20a-5p may regulate these five differences. Metabolites are involved in metabolic pathways to promote the occurrence of PCOS. However, the specific mechanism by which miRNA regulates metabolic pathways and affects that PCOS still needs to be further studied in cell and animal experiments.

Taken together, our results indicated that exosomal miRNAs from PCOS follicular fluid were involved in the regulation of possible pathways, biological functions, and cellular components of PCOS. Moreover, our study constructed miRNA–lncRNA regulatory networks in follicular fluid exosomes, which have crucial biological roles in the occurrence and development of PCOS. Finally, we revealed differences in PCOS metabolism through metabolomic studies. This study is of great significance in revealing new mechanisms of polycystic ovary syndrome and suggesting possible therapeutic targets.

## Figures and Tables

**Table 1:** Information of validated miRNAs.

Primer name	Primer sequence
UR	CAGTGCGTGTCGTGGAGT
h-u6-F	CTCGCTTCGGCAGCACA
h-u6-R	AACGCTTCACGAATTTGCGT
has-miR-34a-5p-RT	GTCGTATCCAGTGCGTGTCGTGGAGTCGGCAATTGCACTGGATACGACACAACCAG
has-miR-34a-5p-F1	TGGCAGTGTCTTAGCTG
hsa-miR-20a-5p-RT	GTCGTATCCAGTGCGTGTCGTGGAGTCGGCAATTGCACTGGATACGACCTACCTG
hsa-miR-20a-5p-F	GCGGGTAAAGTGCTTATAGTGC
hsa-miR-196a-3p-RT	GTCGTATCCAGTGCGTGTCGTGGAGTCGGCAATTGCACTGGATACGACCTCAGGCA
hsa-miR-196a-3p-F1	CGGCAACAAGAAACTGC
hsa-miR-143-5p-RT	GTCGTATCCAGTGCGTGTCGTGGAGTCGGCAATTGCACTGGATACGACACCAGAGA
hsa-miR-143-5p-F3	GGTGCAGTGCTGCA
hsa-miR-106a-3p-RT	GTCGTATCCAGTGCGTGTCGTGGAGTCGGCAATTGCACTGGATACGACGTAAGAAG
hsa-miR-106a-3p-F1	CTGCAATGTAAGCACTT

**Table 2:** Information of PCOS patients and non-PCOS donors.

Parameter	PCOS group (*n* = 15)	Non-PCOS group (*n* = 15)	*P* value
Age	33.2 ± 2.4	30.4 ± 2.9	0.17
Infertility	2.4 ± 1.6	2.6 ± 1.6	0.84
BMI	23.402 ± 2.7	21.984 ± 1.7	0.39
FBG (mmol/L	4.85 ± 0.4	4.632 ± 0.3	0.41
E2 (pg/mL)	49.8 ± 13.3	45.6 ± 12.6	0.66
Progesterone (ng/mL)	0.332 ± 0.13	0.46 ± 0.34	0.51
Testosterone (ng/mL)	0.502 ± 0.12	0.454 ± 0.14	0.63
FSH	5.704 ± 1.03	7.114 ± 0.8	0.07
PRL	10.788 ± 1.6	12.278 ± 3.1	0.41
LH	7.784 ± 3.8	4.992 ± 2.1	0.23
Number of follicles	25 ± 5.9	12.4 ± 5.2	0.01
AMH (ng/mL)	5.278 ± 2.1	1.94 ± 0.5	0.02

Abbreviations: BMI: body mass index; FBG: fasting blood glucose; LH: luteinizing hormone; AMH: anti-Mullerian hormone; PRL: serum prolactin; E2: estradiol; FSH: follicle-stimulating hormone;.

**Table 3:** The expression of miRNAs in the miRNA–lncRNA coexpression network in metabolic pathways.

miRNA	logFC	*P* value	DEG
hsa-miR-32-3p	6.254050282	5.30E-05	up_regulated
hsa-miR-196a-3p	4.452656099	0.000452	up_regulated
hsa-miR-199a-5p	3.455170178	0.000694	up_regulated
hsa-miR-143-5p	3.350954732	0.001784	up_regulated
hsa-miR-26a-2-3p	3.256636073	0.0036	up_regulated
hsa-miR-21-5p	2.052289441	0.008091	up_regulated
hsa-miR-106a-3p	5.883950978	0.010317	up_regulated
hsa-miR-132-3p	1.840853713	0.01079	up_regulated
hsa-miR-125b-1-3p	2.656313696	0.011578	up_regulated
hsa-miR-15a-3p	2.743713774	0.013771	up_regulated
hsa-miR-19a-3p	1.906020762	0.014684	up_regulated
hsa-miR-299-3p	2.111979855	0.017976	up_regulated
hsa-miR-24-1-5p	2.479764956	0.022368	up_regulated
hsa-miR-369-3p	2.247301418	0.023098	up_regulated
hsa-miR-30e-5p	1.467721548	0.023425	up_regulated
hsa-miR-139-5p	2.089759187	0.026395	up_regulated
hsa-miR-129-1-3p	3.7326463	0.028161	up_regulated
hsa-miR-34a-5p	1.607443155	0.028537	up_regulated
hsa-miR-369-5p	2.00127731	0.028573	up_regulated
hsa-miR-371a-3p	3.85268939	0.032162	up_regulated
hsa-miR-181b-2-3p	5.270233934	0.032403	up_regulated
hsa-miR-23a-5p	1.507630692	0.03562	up_regulated
hsa-miR-376c-3p	1.639249701	0.040714	up_regulated
hsa-miR-19b-3p	1.724336734	0.041056	up_regulated
hsa-miR-20a-5p	1.308580728	0.042476	up_regulated
hsa-miR-101-5p	1.6811069	0.045046	up_regulated
hsa-miR-376a-5p	2.293758046	0.046278	up_regulated
hsa-miR-199a-3p	1.52738784	0.047854	up_regulated

## Data Availability

All data can be obtained through the corresponding author.

## Abbreviations

PCOS: Polycystic ovary syndrome
BMI: Body mass index
SHBG: Sex hormone-binding globulin
NF-*κ*b: Nuclear factor-*κ* B
FBG: Fasting blood glucose.

## Supplementary Materials

Supplementary Materials Supplementary Table 1. Antibody information.
