# Supplementary material for: Combined Transcriptomic and Metabolomic Analysis of Women with Polycystic Ovary Syndrome
Source: Dis Markers. 2022 Aug 28;2022:4000424. doi: 10.1155/2022/4000424 (PMC9441417; doi:10.1155/2022/4000424)
Supplement: Supplementary Materials — Supplementary Table 1. Antibody information. [file 4000424.f1.docx]

Supplementary Table 1. Antibody information

| **Antibody** | **Dilution factor** | **article number** |
| --- | --- | --- |
| HSP70 primary antibody | 1:2000 | 10995-1-AP |
| TSG101 primary antibody | 1:2000 | ab125011 |
| Calnexin primary antibody | 1:2000 | ab75801 |
| Rabbit secondary antibody | 1:2000 | 7074S |
